# Erythema induratum of Bazin

**DOI:** 10.1590/0037-8682-0465-2022

**Published:** 2023-02-20

**Authors:** Nurimar Conceição Fernandes, Ana Paula Hortêncio

**Affiliations:** 1 Universidade Federal do Rio de Janeiro, Hospital Universitário Clementino Fraga Filho, Serviço de Dermatologia, Rio de Janeiro, RJ, Brasil.; 2 Universidade Federal do Rio de Janeiro, Hospital Universitário Clementino Fraga Filho, Serviço de Dermatologia, Programa de Residência Médica em Dermatologia, Rio de Janeiro, RJ, Brasil.

**FIGURE 1:**
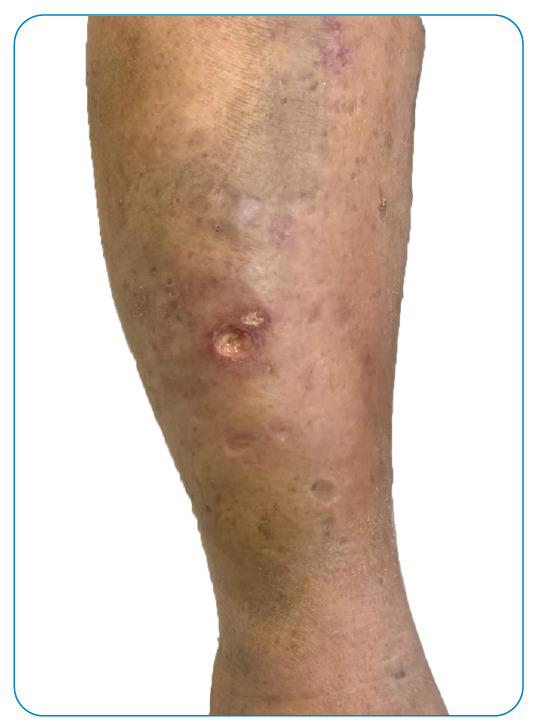
Ulcers with well-defined edges and surrounding erythema, covered by fibrin on the lower leg.

**FIGURE 2:**
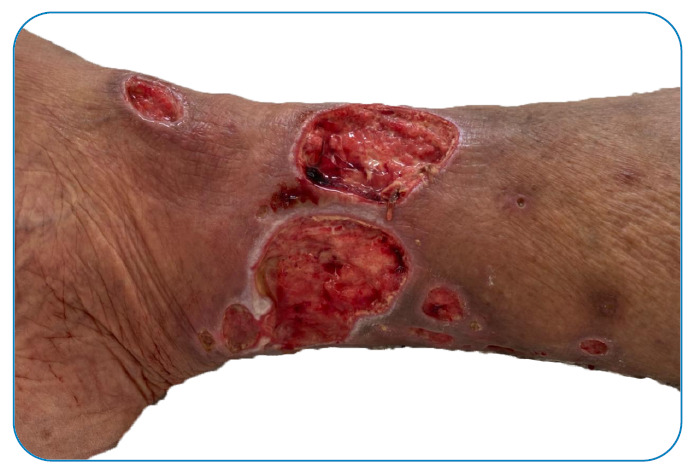
Clean ulcers over indurated plaques after antibiotic therapy on the lower limb.

Here we present the case of a 70-year-old female born in Rio de Janeiro with a histopathological clinical diagnosis made September, 2018, of non-endemic pemphigus foliaceus. Treatment with prednisone (1mg/kg/day) with remission. In 2021, asymptomatic ulcers appeared on the patient’s right lower limb in the areas indurated due to panniculitis (Figures 1, 2). Clinical-epidemiological data of age, gender, venous insufficiency, obesity, and immunodepression supported the diagnosis of erythema induratum of Bazin (EIB).

A fragment of ulcer biopsy in the histopathology showed a granulomatous dermohypodermitis with tissue necrosis and the presence of alcohol-acid resistant bacilli (AARB) by WADE stain and a bacilloscopy negative for AARB by Ziehl-Nielsen stain. The culture result was negative for mycobacteria; however, an ulcer swab for GenExpert detected *Mycobacterium tuberculosis*.

EIB affects mainly middle-aged obese women that have venous insufficiency. Recurrent, violaceous, ulcerated, and painful nodules on the calves are the cutaneous manifestations^1^ ^,^ ^2^. The association with tuberculous infection has been demonstrated by polymerase chain reaction (PCR) amplification for *M. tuberculosis* DNA in skin biopsy specimens^1^ ^,^ ^2^.

The GenExpert® MTB/RIF test is useful to distinguish tuberculosis from infections with atypical *Mycobacterium*. A positive result does not necessarily indicate the presence of viable organisms. In cutaneous tuberculosis, the specificity/sensitivity in the different tests are as follows: PCT GenExpert (98.4%/69%), culture (98%/23%), bacilloscopy (100%/14%)^3^.

Misdiagnosis of EIB in daily practice is likely, as cutaneous tuberculosis can mimick several dermatoses. EIB may need longer treatment periods, mainly in cases of recurrent episodes^2^.
